# Comparison of the regenerative potential of different functionalized gelatin-based hydrogels as fillers of rabbit corneal wounds

**DOI:** 10.3389/fmed.2025.1667446

**Published:** 2025-09-24

**Authors:** Cristina Romo-Valera, Jaime Etxebarria, Vanesa Freire, Maddalen Rodriguez-Astigarraga, Jon Arluzea, Noelia Andollo

**Affiliations:** ^1^Department of Cell Biology and Histology, School of Medicine and Nursing, University of the Basque Country UPV/EHU, Leioa, Spain; ^2^BEGIKER Ophthalmology Research Group, IIS Biobizkaia, Barakaldo, Spain; ^3^Department of Ophthalmology, University Hospital of Cruces, Barakaldo, Spain; ^4^R&D&I Department, ICQO Instituto Clínico-Quirúrgico de Oftalmología, Bilbao, Spain

**Keywords:** gelatin, riboflavin-phosphate, functionalized-hydrogels, corneal wound healing, persistent epithelial defects, corneal regeneration

## Abstract

**Objectives:**

Persistent epithelial defects (PEDs) and chronic corneal ulcers are lesions resistant to treatment for over 2 weeks, risking inadequate healing, reduced sensitivity, and corneal lysis or perforation. This study evaluates the regenerative potential of functionalized gelatin-based hydrogels for treating rabbit corneal wounds as a non-surgical alternative.

**Methods:**

Thirty female New Zealand white rabbits underwent anterior stromal keratectomy and were assigned to five groups: control (0.2% HA artificial tears) and four hydrogel treatment groups. Hydrogels included non-functionalized gelatin-RFP (H) and functionalized versions with infliximab (H-Ab), autologous serum (H-AS), and human amniotic membrane extracts (H-HAMe). Crosslinking was performed *in situ* with blue light. Corneas were evaluated at 7 and 21 days for re-epithelialization, fibrosis, and inflammation using histology, qPCR and immunohistochemistry, focusing on markers of proliferation (Ki67), differentiation (CK3), stemness (*PAX6, p63, CD44*), adhesion (integrin β4), and fibrosis (*α*-SMA).

**Results:**

All treatments supported re-epithelialization by day 7 and restored barrier function (ZO-1), with H-AS achieving the fastest closure. Expression of the adhesion marker integrin β4 improved over time across all groups. Hydrogel formulations promoted limbal activation (*PAX6, CD44*), with H-AS and H-HAMe showing elevated *p63* expression at day 7. All hydrogels reduced fibrosis (*α*-SMA), though extracellular matrix organization varied. H-Ab and H-HAMe reduced inflammation (*IL-1β*), while H-AS showed minimal irritability.

**Conclusion:**

Functionalized gelatin-RFP hydrogels promote re-epithelialization, reduce fibrosis and inflammation, and restore ocular integrity, offering a promising solution for corneal wound repair.

## 1 Introduction

The human cornea is a transparent, avascular tissue that accounts for approximately two-thirds of the eye’s total refractive power. Despite its critical function, it is particularly vulnerable to environmental insults such as trauma, infections, and chemical injuries (1). Damage to the corneal epithelium can result in persistent epithelial defects (PEDs) and corneal ulcers, which ulcers represent significant therapeutic challenges in ophthalmological practice, frequently resulting in severe complications such as scarring, corneal perforation and irreversible visual loss (2, 3). While standard therapies—such as lubricants, antibiotics, and eye drops enriched with specific bioactive compounds—can offer symptomatic relief, they often fail to adequately promote tissue regeneration, particularly in severe or chronic cases.

Recent advances in regenerative medicine have transformed the therapeutic landscape, particularly through the integration of biomaterials with biologically active and blood-derived agents. These strategies have opened new avenues for promoting corneal healing and restoring visual function (4–8). Hydrogels have emerged as promising platforms for corneal repair, offering biocompatible, tunable scaffolds capable of delivering therapeutic agents in a sustained and localized manner (9). Both natural and synthetic hydrogels—including gelatin (10–12), collagen (13, 14), chitosan (15, 16), polyethylene glycol (PEG) (17, 18), and hyaluronic acid (HA) (13, 19)—have been explored for their potential to support epithelial regeneration.

Among the most extensively studied biological therapies are blood-derived products, which contain a complex array of growth factors and proteins at concentrations similar to those found in natural tear fluid. These constituents are vital for promoting epithelial repair and maintaining ocular surface homeostasis. Autologous serum (AS) eye drops—pioneered as the first blood-derived treatment for ocular surface diseases—deliver more than lubrication; they provide essential bioactive molecules such as vitamin A, epidermal growth factor (EGF), and fibronectin, all of which support epithelial proliferation and differentiation (20–22). In parallel, plasma rich in growth factors (PRGF) eye drops have demonstrated effectiveness in conditions such as neurotrophic keratitis, dry eye disease, and post-surgical corneal wound healing, including after photorefractive keratectomy and epithelial debridement (23–31).

The therapeutic potential of the human amniotic membrane (AM) and its derivatives is also well established. Acting as both a structural scaffold and a reservoir of bioactive factors, AM supports epithelial cell proliferation and migration while exhibiting anti-inflammatory, immunomodulatory, antibacterial, anti-scarring, hemocompatible, and angio-modulatory properties (32–37). Like AS and PRGF, AM-derived products deliver a rich combination of cytokines and growth factors that suppress inflammation and promote corneal healing. Nevertheless, their broader clinical application has been hindered by variability in biological composition and the lack of effective sustained delivery mechanisms.

Combining these biological agents with hydrogel systems represents a promising strategy for overcoming these limitations. Namely, the incorporation of anti-inflammatory molecules into hydrogels has been shown to enhance corneal healing outcomes by mitigating inflammation and preventing ulcer formation (38). Infliximab, a monoclonal antibody targeting tumor necrosis factor alpha (TNF-*α*), has demonstrated protective effects in dry eye disease and corneal neovascularization, and is currently being investigated for use in treating corneal melt (39–42).

In our previous work, we developed a photo-crosslinkable hydrogel composed of 5% gelatin and 0.01% riboflavin phosphate (RFP), crosslinked using blue light. This system was thoroughly characterized *in vitro* and *ex vivo* for applications in corneal tissue engineering, demonstrating favorable gelation kinetics, mechanical resilience, cytocompatibility, and the ability to support epithelial cell integration and migration (43). Building upon these foundational results, the present study extends our research into an *in vivo* context, employing a rabbit stromal keratectomy model to evaluate the therapeutic efficacy of gelatin–riboflavin hydrogels functionalized with autologous serum (AS), human amniotic membrane extract (HAMe), or infliximab. The *in vivo* model allows for a more comprehensive assessment of ocular healing, closely mimicking clinical conditions and enabling the evaluation of both early and sustained responses to treatment. Specifically, we monitored ocular morbidity, wound healing progression, gene expression profiles, and immunohistochemical analyses to investigate the modulation of inflammation, fibrosis, and re-epithelialization. This study provides a systematic comparison that simultaneously evaluates three distinct bioactive agents usually used in clinical ophthalmology (autologous serum, infliximab, and HAMe) integrated into the same hydrogel platform. The idea is to contribute to the advancement of ocular regenerative medicine through an innovative approach and a comprehensive comparative methodology.

## 2 Materials and methods

### 2.1 Hydrogel synthesis

Porcine skin gelatin (10% w/v, Sigma Aldrich, St. Louis, MO, United States) was crosslinked with riboflavin phosphate (RFP, 0.02% w/v; Sigma Aldrich) to synthesize hydrogels. Infliximab-loaded hydrogels were prepared by mixing infliximab (2 mg/mL, Merck KGaA, Darmstadt, Germany) with gelatin-RFP stock to obtain final concentrations of 1 mg/mL infliximab, 5% w/v gelatin, and 0.01% w/v RFP. Hydrogels with autologous serum (AS) or HAMe were prepared by combining gelatin-RFP stock with AS or HAMe extracts (1,1 dilution), resulting in 50% of AS or 50% HAMe, 5% w/v gelatin, and 0.01% w/v RFP. All hydrogels were adjusted to pH 7 with 1 M NaOH, filtered (0.22 μm, MILLEX® filters, Merck KGaA), and stored at 4 °C.

To prevent photodegradation, hydrogel solutions were shielded from light by storing them in amber glass containers or covering the vessels with aluminum foil during both the mixing process and storage. Once the wounds were filled with the hydrogels, they were crosslinked for 2 mins under blue light using the Led.C curing lamp (Woodpecker Medical Instrument Co., Guilin, Guangxi, China), which operates within a wavelength range of 420–480 nm and a light intensity of 1,000–1,200 mW/cm^2^. To ensure consistent exposure and reproducibility, the lamp was maintained at a fixed distance of 10 cm from the animals.

Detailed *in vitro* physicochemical characterizations of the gelatin–riboflavin hydrogel, including rheology, optical transparency, swelling behavior, and release kinetics, were comprehensively reported previously (43).

### 2.2 Human amniotic membrane extract preparation

Placentas were obtained with informed consent, following the Declaration of Helsinki and Ethics Committee approval (University Hospital of Cruces). The amnion was manually separated from the chorion and the HAM was sectioned into distal, medial, and proximal regions relative to the placenta. Sections were placed in sterile DMEM (Lonza Bioscience, Basel, Switzerland) supplemented with 1.25 μg/mL amphotericin B (Gibco, Paisley, United Kingdom), 50 μg/mL penicillin–streptomycin (Sigma Aldrich), and 50 μg/mL neomycin (Gibco), cut into 4–4.5 cm pieces, washed, and stored at −80 °C until use. To obtain HAM extracts, HAM fragments were frozen in liquid nitrogen for 5 min, powdered in a pre-cooled mortar, weighed, and resuspended in PBS with 5 mL/g protease inhibitor (P8340, Sigma Aldrich). Samples were sonicated for 20 min at 90% amplitude (Bandelin Sonoplus, Sigma Aldrich) using a 1.5 mm probe, centrifuged (3,000 g, 10 min, 4 °C), and supernatants were collected. The solution was filtered (0.22 μm MILLEX® filters), aliquoted into 0.2 mL tubes, and stored at −80 °C.

### 2.3 Animals

Thirty 2-kg female New Zealand white rabbits were used for *in vivo* studies. Experiments adhered to protocols approved by the Animal Research Ethics Committee of the University of the Basque Country (UPV/EHU) and complied with the Tenets of the Declaration of Helsinki and the ARVO Statement for the Use of Animals in Ophthalmic and Vision Research. All procedures were conducted at the UPV/EHU animal facility (Sgiker).

### 2.4 Extraction of rabbit autologous serum

After locally anesthetizing the rabbit with lidocaine, the blood was collected by central ear artery venipuncture into Vacutainer® tubes containing a polymer gel for serum separation (BD, Franklin Lakes, NJ, United States).

Blood was allowed to clot for 2 h at room temperature and centrifuged at 1,000 g for 15 min. The collected supernatants, which constituted the AS, were filtered (0.22 μm MILLEX® filters) and stored at −80 °C until their use.

### 2.5 Surgical procedure

Thirty rabbits were divided into five groups (*n* = 6 each): a control group treated with 0.2% HA artificial tears (Dr. Gerhard Mann, Chem.-pharm. Fabrik GmbH, Berlin, Germany) and four groups treated with gelatin-RFP hydrogels: non-functionalized (H) or functionalized with infliximab (H-Ab), autologous serum (H-AS), or human amniotic membrane extract (H-HAMe). The four groups treated with hydrogel also received 0.2% HA artificial tears.

The rabbits were further divided into three surgery groups (*n* = 10 each). Surgery was performed first on the right eye, followed by a 1-week washout period before repeating the procedure on the left eye. A pilot study involving 10 eyes established experimental conditions.

**Figure 1:**
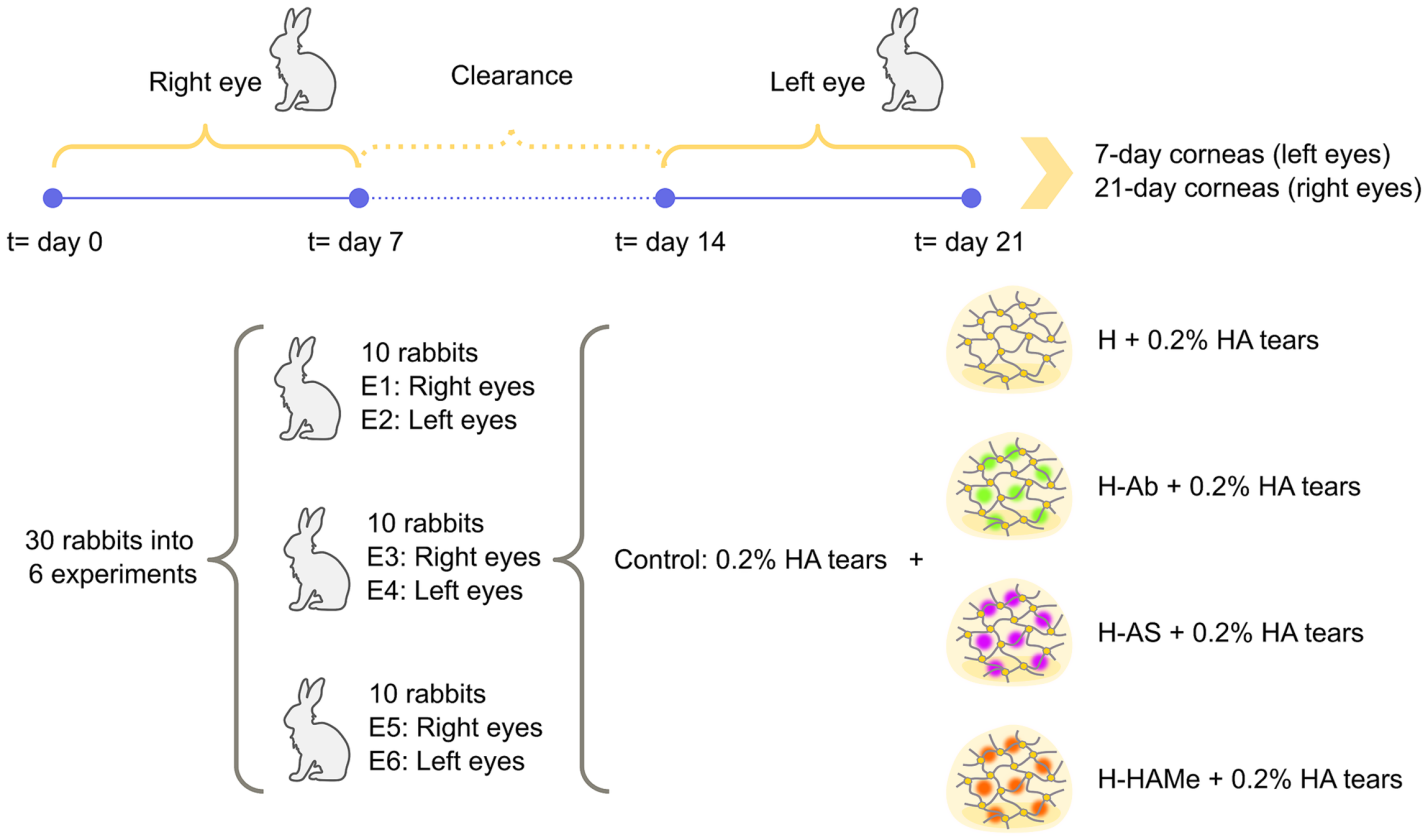
Outline of experimental procedure. 30 female New Zealand white rabbits were divided into 5 groups: 4 study groups (H, H-Ab, H-AS, H-HAMe) and a control group, and 6 experiments. Odd experiments (E01/E03/E05) used right eyes and even experiments (E02/E04/E06) used left eyes. Control eyes received surgery and 0.2% HA artificial tears 4 times daily until wound closure, also given to hydrogel-treated corneas. Surgery was first done on right eyes (2 per group), treated, and evaluated for 7 days. After a 1-week clearance, the procedure was repeated on left eyes. Both corneas from each rabbit were collected on day 21 for histology and inflammation analysis.

In each surgery round, the right eyes of the 10 rabbits were operated on first. These eyes were allocated into the treatment and control groups (two eyes per treatment) and monitored over 7 days. After this period, a 7-day washout interval was observed to ensure clearance of any residual medication. On day 14 (counting from the right-eye surgery), the left eyes of the same rabbits were operated on. Treatments for the left eyes were assigned randomly, and both eyes of a given rabbit did not necessarily receive the same treatment. The left eyes were monitored for an additional 7 days. On day 21 of the cycle (relative to the right-eye surgery), all animals were euthanized, and both corneas were collected. At that time, right eyes had been followed for 21-days after right-eye surgery whereas left eyes had been followed for only 7-days after left-eye surgery. This procedure was repeated for two additional rounds, resulting in a total of 30 rabbits and 60 eyes across all experiments. Experiments 1, 3, and 5 corresponded to right eyes; Experiments 2, 4, and 6 corresponded to left eyes. An outline of the experimental development is shown in Figure 1 while the complete experimental design can be found in Supplementary Table S1.

Anesthesia was administered intramuscularly using 1 mL/kg ketamine (Ketolar® 50 mg/mL, Pfizer, Spain) and 0.3 mL/kg xylazine (Xylagesic 20 mg/mL, Laboratorios Calier, Spain). After applying double anesthesia eye drops (oxybuprocaine and tetracaine, Alcon Healthcare, Spain), a 6.5 mm corneal Hessburg-Barron trephine (Katena, CorzaMedical, United States) created an anterior stromal keratectomy (3/4 turn, 187 μm depth), followed by defect excision with a crescent blade. The defect was filled with 50 μL preheated hydrogel at 34 °C, crosslinked *in situ* with blue light (Led.C curing lamp, Woodpecker Medical Instrument Co, China) for 2 min and protected by partial tarsorrhaphy for 72 h. Postoperative care included 0.05 mg/kg subcutaneous buprenorphine (Buprecare®, Ecuphar, Spain) every 12 h for 72 h, Tobrex® (Tobramycin, Novartis, Spain) antibiotic eye drops (2 × daily) and HA artificial tears (4 × daily) until closure. Eyes were assessed daily for 7 days. Euthanasia was performed using 2 mL/kg ketamine, 0.6 mL/kg xylazine, and intravenous saturated potassium chloride, with death confirmed by rigor mortis.

### 2.6 Clinical evaluation of eye injuries

Ocular morbidity was evaluated from days 3 to 7 using the Draize ocular toxicity test via slit-lamp examination. This test scores corneal opacity, iritis, conjunctival redness, edema, and discharge, with total scores (0–110) classified per Kay and Calandra criteria (44).

Iris and conjunctiva were examined first, followed by corneal assessment using 5 μL of 2% fluorescein Colicursí® Fluotest eye drops (Novartis) under blue light. Photographs were taken on day 0 and from day 3 on until wound closure. The fluorescein-stained area was quantified with ImageJ software. On day 7 (left eyes) or day 21 (right eyes), corneas were dissected and prepared for further histology, immunostaining or gene expression analysis.

### 2.7 RNA extraction, retrotransciption, cDNA preamplification and real-time PCR

RNA was extracted from corneal tissues using a bullet blender (Next Advanced, NY, United States) with zirconium oxide beads and Trizol (Thermo Fisher Scientific, Waltham, MA, United States) following the manufacturer’s protocol. Extracted RNA was treated with DNase I (1 unit/μl; Zymo Research, Irvine, CA, United States) for 15 min at room temperature, followed by inactivation with EDTA (2.5 μL, 25 mM) and heating at 65 °C for 10 min.

Reverse transcription of 1 μg RNA per sample was performed using the iScriptTM cDNA Synthesis Kit (Bio-Rad, Hercules, CA, United States). cDNA pre-amplification was carried out with the Multiplex PCR Kit (Qiagen, Hilden, Germany) under thermal conditions of 95 °C for 15 min, 14 amplification cycles, and a 30-min extension at 60 °C, including in the pre-amplification mix the primers to be used later.

qPCR was performed using a CFX96 Real-Time System (Bio-Rad) with SYBR® Green dye. Reactions (20 μL) included 1 μL pre-amplified cDNA, 250 nM gene-specific primers, and iQ SYBR Green Supermix (Bio-Rad). The protocol comprised polymerase activation (95 °C, 3 min), 40 cycles of denaturation (95 °C, 30 s), annealing (gene-specific temperatures, 30 s), and elongation (72 °C, 30 s). Melting curves (65–95 °C in 0.5 °C increments over 60 cycles) were generated post-amplification, followed by a 4 °C hold. Primer details are in Table 1.

**Table 1:** Sequences, amplicon sizes and annealing temperatures (Ta) of the primers used in *in vivo* assays.

Primer	Type	Sequence (5′ → 3′)	Amplicon size	Ta
*CD44*	Fwd.	GACACCATGGACAAGTTTTGG	140	59.8
	Rev.	GAGATGCTGTAGCGACCATT		
*CK3*	Fwd.	GACTCGGAGCTGAGAAGCAT	198	59.8
	Rev.	CAGGGTCCTCAGGAAGTTGA		
*IL-1β*	Fwd.	GTAGACCCCAACCGTTACCC	145	56.4
	Rev.	AGACGGGCATGTACTCTGTC		
*Ki 67*	Fwd.	GCCAAGATAGTTGCTGATAC	171	58.4
	Rev.	AAGTGTCCGATTCCGATTA		
*p63*	Fwd.	CGCCCCTTTCGTCAGAACAC	165	57.2
	Rev.	GTGCTGAGGAAGGTACTGCAT		
*PAX6*	Fwd.	AGAGAATACCAACTCCATCAG	152	58.4
	Rev.	GATAATGGGTTCTCTCAAACTC		
*α-SMA*	Fwd.	CGGTGCTGTCTCTCTATGCC	177	58.4
	Rev.	CACGCTCAGTCAGGATCTTCA		
*GADPH*	Fwd.	TCGGAGTGAACGGATTTG	225	61
	Rev.	CTCGCTCCTGGAAGATGG		
*HPRT1*	Fwd.	ACGTCGAGGACTTGGAAAGGGTGTT	96	58.4
	Rev.	GGCCTCCCATCTCCTTCATCACATC		
*RIG S15*	Fwd.	CATGGTGGGCGTCTACAAC	161	58.4
	Rev.	ACTTGAGAGGGATGAAGCGG		

Negative and RT-minus controls were included. Samples at 7 and 21 days were analyzed with 3 and 2 biological replicates, respectively, and 3 technical replicates each. Detection ranges were assessed with 5-fold serial dilutions (1:5 to 1:625), achieving over 99% PCR efficiency and R2 above 0.98 for all primers. Expression was normalized using *GAPDH*, *HPRT1*, and *RIG/S15* as reference genes, and data were analyzed via Bio-Rad CFX Maestro software.

### 2.8 Histological examination

Frozen corneal sections of 10 μm thickness were obtained from OCT-embedded tissues using a CM 3050S cryostat (Leica, Wetzlar, Germany). Following sectioning, the samples were fixed in 4% paraformaldehyde (PFA), stained with H&E, mounted with Dibutylphthalate polystyrene xylene (DPX) under coverslips and observed with an Olympus BX50 microscope (Olympus, Tokyo, Japan).

### 2.9 Immunohistochemistry

Frozen 10 μm slides were thawed and rinsed twice with PBS (5 min each). Sections were permeabilized with 0.1% Triton TX-100 (Sigma Aldrich) in PBS (PBST) (10 min, twice), blocked with 10% normal goat serum (Thermo Fisher Scientific) (10 min), and incubated overnight at 4°C with primary antibodies (Table 2) in blocking solution (0.1% BSA, 5% FBS in PBST).

**Table 2:** List of primary and secondary antibodies used for immunofluorescence assays.

Inmmunogen	Reference	Company	Isotype	Dilution	Type
CK15	sc-47697	Santa Cruz	IgG2a	1:50	Primary
CK3/K76	CBL218	Millipore	IgG1	1:50	Primary
Integrin β4	ab29042	Abcam	IgG1	1:40	Primary
Ki67	MAB4190	Millipore	IgG1	1:40	Primary
ZO-1	ab190085	Abcam	IgG	1:40	Primary
α-SMA	ab7817	Abcam	IgG2a	1:400	Primary
anti-Goat IgG	A11057	ThermoFisher scientific	IgG	1:1,000	Secondary
anti-Rabbit IgG	ab175471	Abcam	IgG	1:1,000	Secondary
anti-Rabbit IgG	A11070	ThermoFisher scientific	IgG	1:1,000	Secondary
anti-Mouse IgG1	A21121	ThermoFisher scientific	IgG	1:1,000	Secondary
anti-Mouse IgG1	A21124	ThermoFisher scientific	IgG	1:1,000	Secondary
anti-Mouse IgG2a	A21131	ThermoFisher scientific	IgG	1:1,000	Secondary
anti-Mouse IgG2a	A21134	ThermoFisher scientific	IgG	1:1,000	Secondary

After PBS (10 min, once) and PBST washes (15 min twice), samples were stained with secondary antibodies (1,1,000 in blocking solution) for 1.5 h at room temperature in the dark, washed again, and counterstained with 4 mg/mL Hoechst 33342 (Thermo Fisher Scientific) (15 min, room temperature). Final PBS washes (5 min, twice) preceded mounting with Fluoromount G (SouthernBiotech; Birmingham, United Kingdom). Slides imaged with a Nikon Ti-U fluorescence microscope (Nikon, Tokyo, Japan).

### 2.10 Analysis and statistics

Data normality was assessed using Shapiro-Wilks tests, confirming nonparametric distribution (Supplementary Tables S2, S3). A Kruskal-Wallis test followed by Dunn’s multiple comparison test was applied in all the results, with significance set at *p* < 0.05. Statistical analyses were conducted using R software (R Foundation for Statistical Computing, Vienna, Austria) and GraphPad Prism 8 (San Diego, CA, United States).

## 3 Results

### 3.1 *In vivo* re-epithelialization in a rabbit animal model

**Figure 2:**
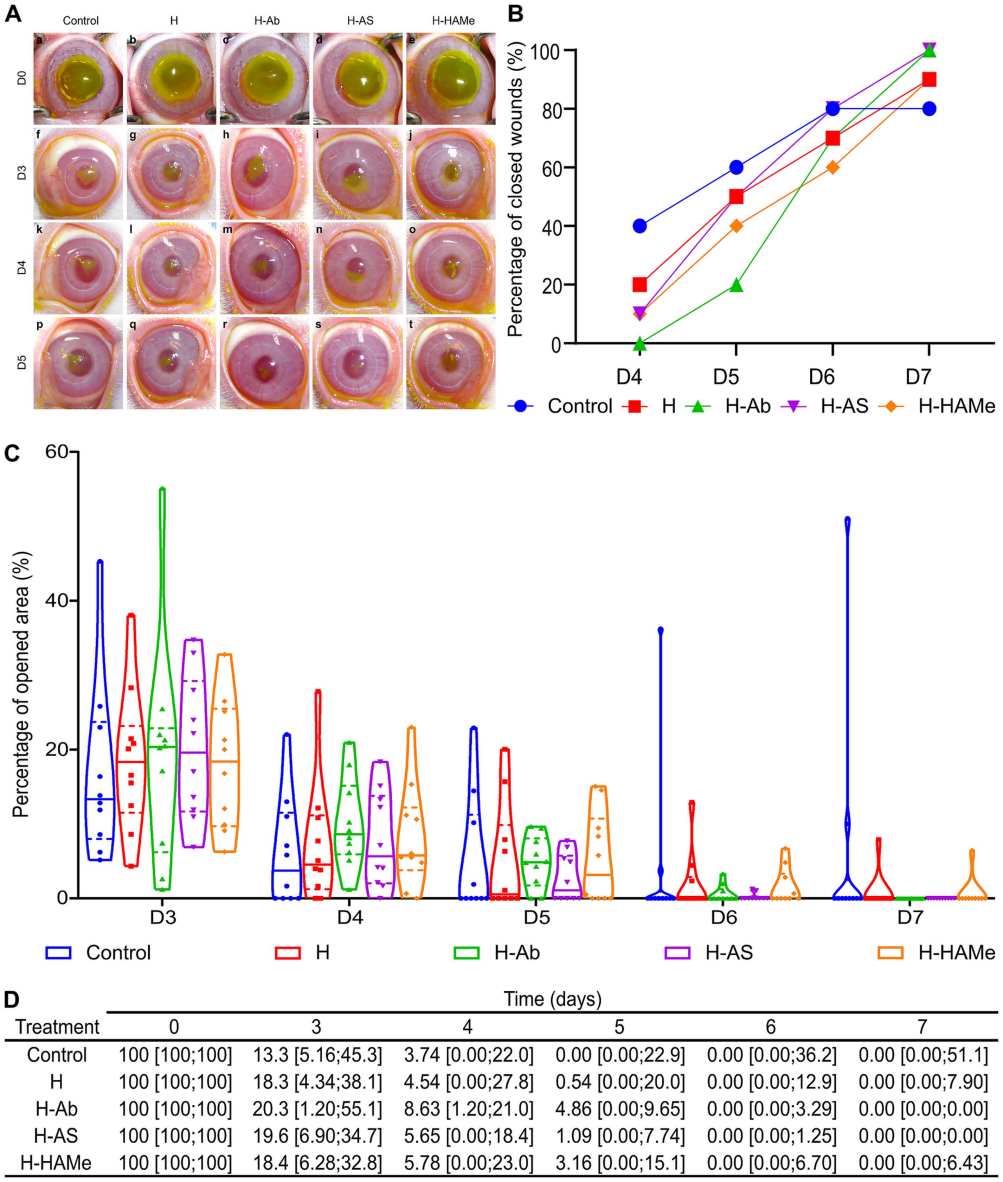
**(A)** Progression of epithelial defects in rabbit eyes treated with artificial tears (Control) or hydrogel variants (H, H-Ab, H-AS, H-HAMe) from day 3 (D3) to day 5 (D5), assessed via fluorescein staining. **(B)** The percentage of wounds closed per day from day 4 (D4) to day 7 (D7). **(C)** Percentage of the surgical wound area remaining open from day 3 (D3) to day 7 (D7), with median (solid line) and interquartile range (dashed line) shown in violin plots. **(D)** Re-epithelialization progress in rabbit eyes treated with hydrogel variants (H, H-Ab, H-AS or H-HAMe) or control, shown as median wound area (%) and minimum and maximum values [min; max]. No significant differences were observed between treatments and samples over time.

Corneal wounds were assessed daily post-tarsorrhaphy removal (day 3). By this time, median wound areas across groups reduced to ≤ 20% (Figures 2A,C). The control group achieved the most rapid closure considering all groups, with 60% of wounds fully closed by day 5 (Figures 2B–D), though 20% remained unhealed by day 7 due to reopening or plateauing (Figures 2A–C).

Hydrogel treatments facilitated steady healing. H-AS promoted the fastest closure among the hydrogel treatments, with a median wound area of 1.09% [0.00; 7.74] by day 5 and complete healing by day 7 (Figures 2B–D). H-Ab-treated corneas exhibited slower early closure, with 4.86% [0.00; 9.65] open on day 4, but all were healed by day 7. H and H-HAMe treatments showed similar trends, achieving 90% closure by day 7, though 10% remained unhealed (Figures 2B–D). Biological variability likely explained the absence of significant differences between groups.

**Figure 3:**
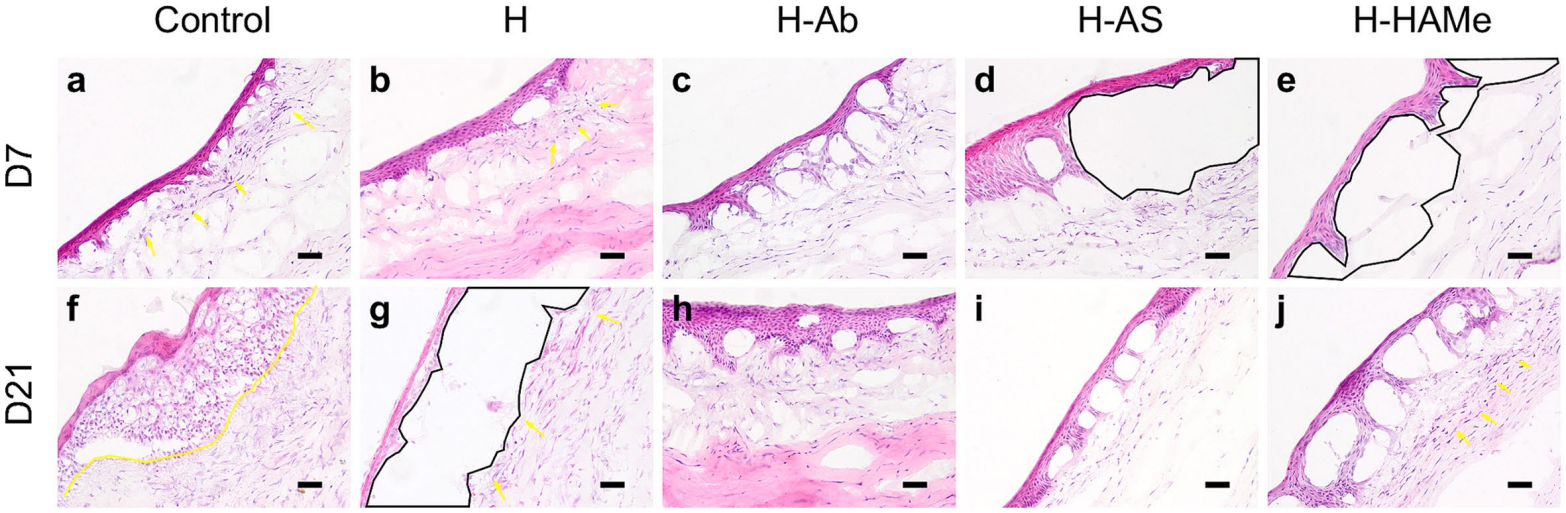
Hematoxylin-Eosin staining of central cornea sections from rabbit eyes post re-epithelialization. Samples treated with artificial tears (Control) or hydrogels (H, H-Ab, H-AS, H-HAMe) were analyzed at 7 **(a–e)** and 21 days **(f–j)** post-surgery. Yellow arrows and highlighted regions indicate myofibroblast infiltration, while black areas denote epithelial detachment. Images at 20 × magnification; scale bars = 50 μm.

Histological analysis confirmed epithelial regrowth across all treatments. Cryoprocessing compromised tissue structure preservation, particularly in the anterior stroma. At 7 days, control corneas showed epithelial thickening and abundance of stromal myofibroblast, progressing to stromal edema and increased myofibroblasts number by 21 days (Figures 3a,f). H-treated corneas displayed epithelial thickening and wound-edge myofibroblasts at 7 days, followed by epithelial thinning and increased myofibroblasts at 21 days (Figures 3b,g). All corneas treated with functionalized hydrogels exhibited a more compact epithelium and robust epithelial-stromal adhesion at 21 days. Epithelial detachment shown at 7 days in H-AS and H-HAMe was likely due to hydrogel loss during tissue processing (Figures 3d,i). By day 21, corneas treated with H-AS exhibited a well-organized extracellular matrix and an absence of myofibroblasts (Figures 3d,j), whereas those treated with H-Ab and H-HAMe showed mild matrix disorganization. Corneas treated with H-Ab displayed a reduced presence of myofibroblasts (Figures 3c,h), while those treated with H-HAMe showed a higher presence of myofibroblasts at the wound margins (Figures 3e,j).

### 3.2 Epithelial cell differentiation, proliferation and stemness

**Figure 4:**
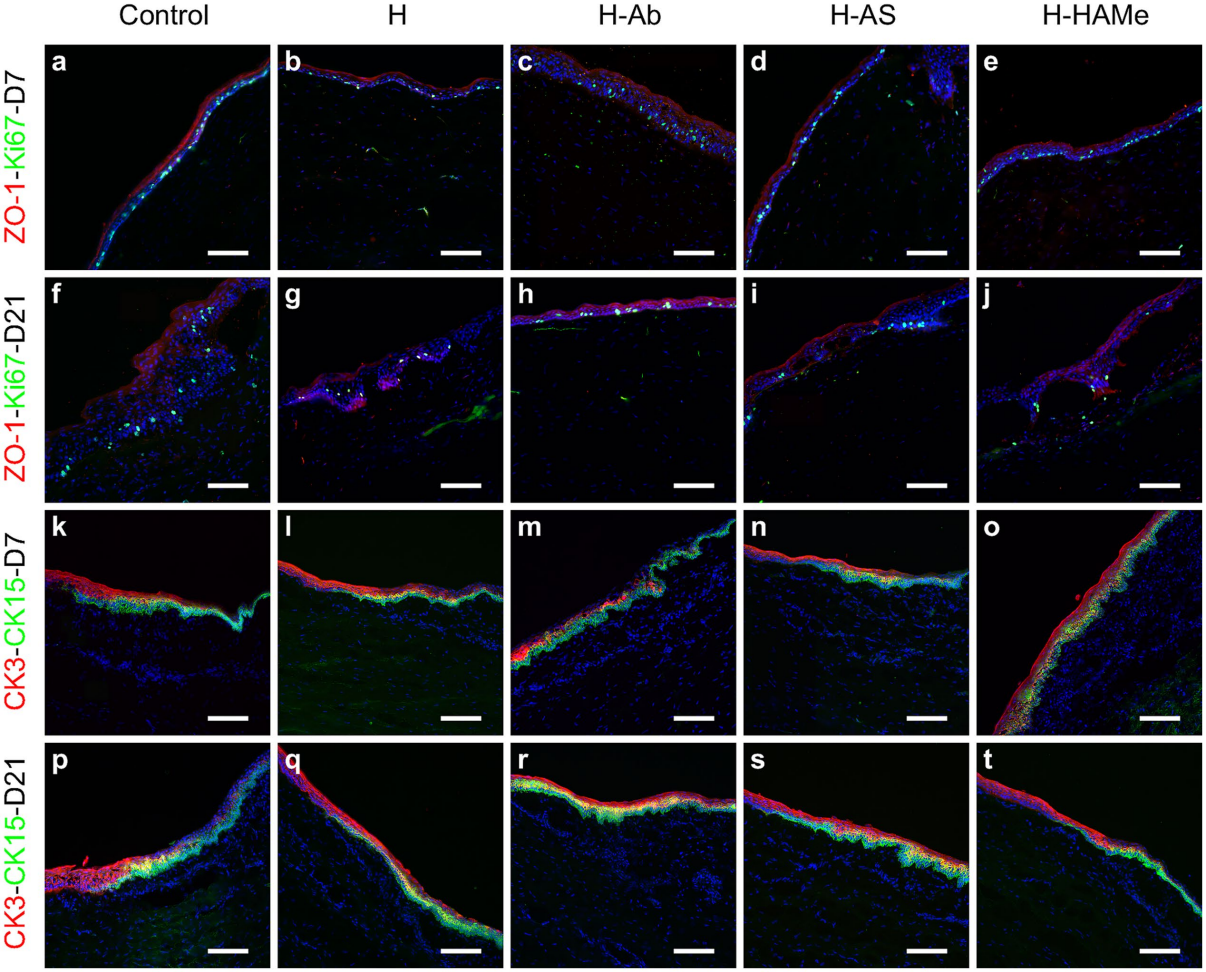
Protein expression of ZO-1 (red), Ki67 (green) **(a–j)** in the central cornea and CK3 (red), CK15 (green) in the limbus **(k–t)** of rabbit corneas treated with artificial tears (Control) or hydrogels (H, H-Ab, H-AS, H-HAMe). Samples analyzed at 7 **(a–e,k–o)** and 21 days **(f–j,p–t)** post-surgery. Images at 20 × magnification; scale bars correspond to 100 μm.

**Figure 5:**
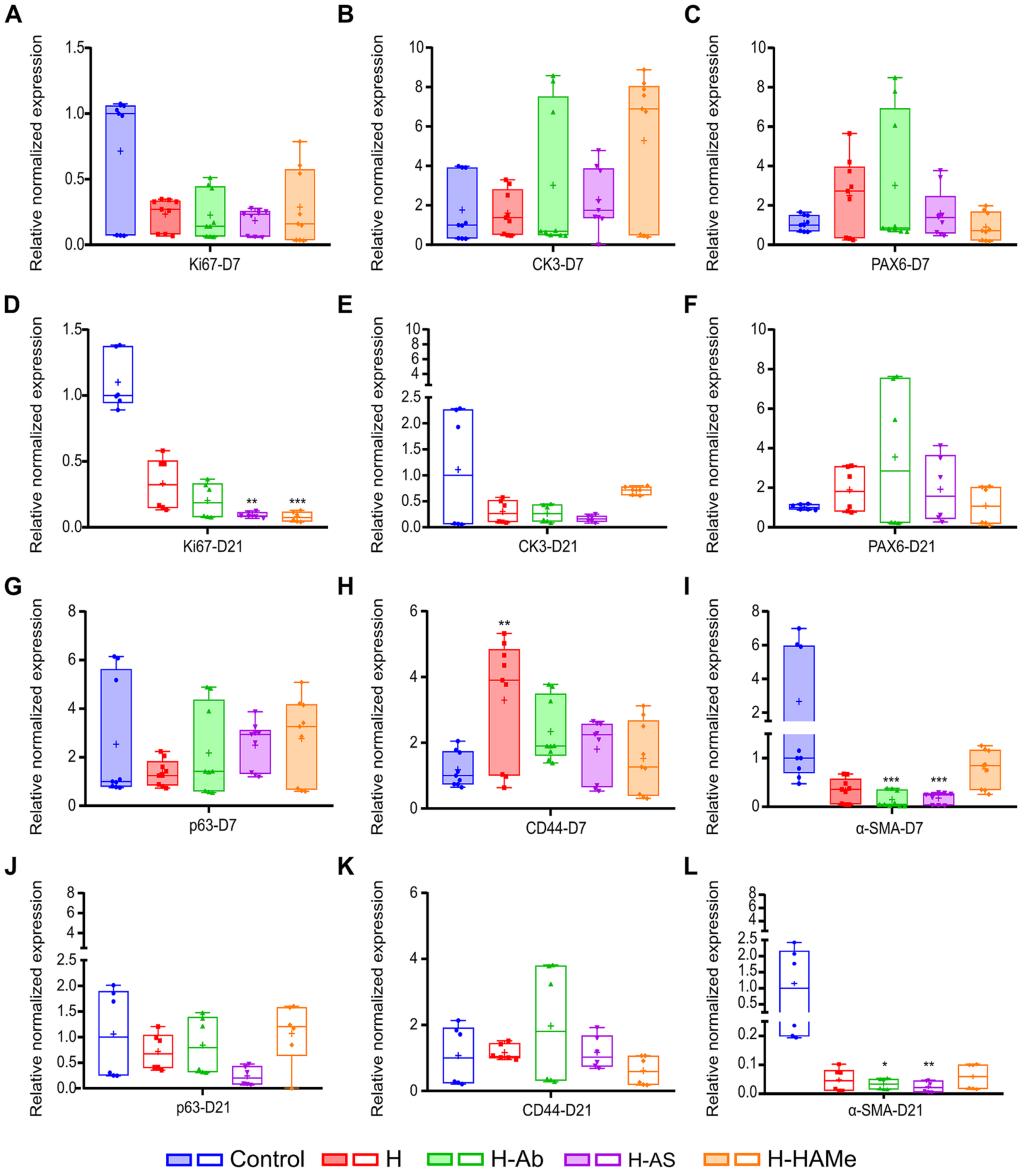
Relative normalized gene expression (ΔΔCq) of *Ki67*, *CK3*, *PAX6*, *p63*, *CD44* and *α-SMA* in rabbit corneas 7 days **(A–C,G–I)** and 21 days **(D–F,J–L)** post-surgery. “+” sign is used to denote the mean value of each box plot. Significant differences (^∗^*p* < 0.05, ^∗∗^*p* < 0.01, ^∗∗∗^*p* < 0.001, ^∗∗∗∗^*p* < 0.0001) are indicated relative to the control treatment for each time point.

ZO-1, a marker of corneal barrier integrity, was consistently detected by immunohistochemistry in the superficial epithelial layers within the wound area across all groups and time points, demonstrating the restoration of epithelial barrier function (Figures 4a–j). In parallel, cell proliferation was evaluated. The highest proliferative activity was observed in the epithelia of untreated controls at both time points (Figures 4a,f). Ki67-positive cells, indicative of cellular proliferation, were most abundant at day 7 in the control group, with a substantial decrease in all treatment groups by day 21 (Figures 4a–j). Consistently, at the RNA level, *Ki67* transcripts were markedly reduced in all hydrogel-treated groups by day 21, with H-AS and H-HAMe showing statistically significant decreases (*p* = 0.0031 and *p* = 0.0008, respectively) compared to controls (Figures 5A,D).

The CK3 differentiation protein marker was centrally expressed in all samples and decreased toward the limbus (Figures 4k–t). CK15 protein labeling, a potential limbal stem cell marker, was restricted to the limbus and co-localized with CK3 in the peripheral cornea. CK15 expression increased by 21 days, indicating limbal activation (Figures 4p–t).

qPCR analysis revealed no significant differences in RNA expression at 7 or 21 days for the differentiated epithelial cells’ marker *CK3* (Figures 5B,E), though H-HAMe had the highest median levels at 7 days, showing 6.89 fold change expression with respect to all the other treatments (Figure 5B). By day 21, *CK3* expression had notably declined in all hydrogel-treated corneas (Figure 5E). H-HAMe reached levels similar to the control, while *CK3* levels in corneas treated with H, H-Ab, and H-AS were markedly lower than those in the control group (Figures 5B,E).

Limbal stem/progenitor markers *PAX6* and *p63* showed no significant differences from controls at RNA expression levels (Figures 5C,F,G,J). Still, *PAX6* transcript levels remained above control levels at day 21 in all H-treated groups, suggesting a promotion of limbal stemness (Figure 5F). *p63* expression peaked at day 7 in H-AS and H-HAMe, but decreased by day 21, while remaining stable in H and H-Ab (Figures 5G,J). Gene expression levels of *CD44*, a transmembrane receptor protein that mediates cell adhesion, migration and stem cell retention in the niche, were elevated in all hydrogel-treated corneas, with H significantly exceeding control levels at 7 days (*p* = 0.0035, Kruskal-Wallis test followed by Dunn’s multiple comparisons) (Figure 5H). By day 21, all hydrogel-treated corneas showed levels equivalent to the control (Figure 5K).

### 3.3 Cell-substrate adhesion and fibrosis

**Figure 6:**
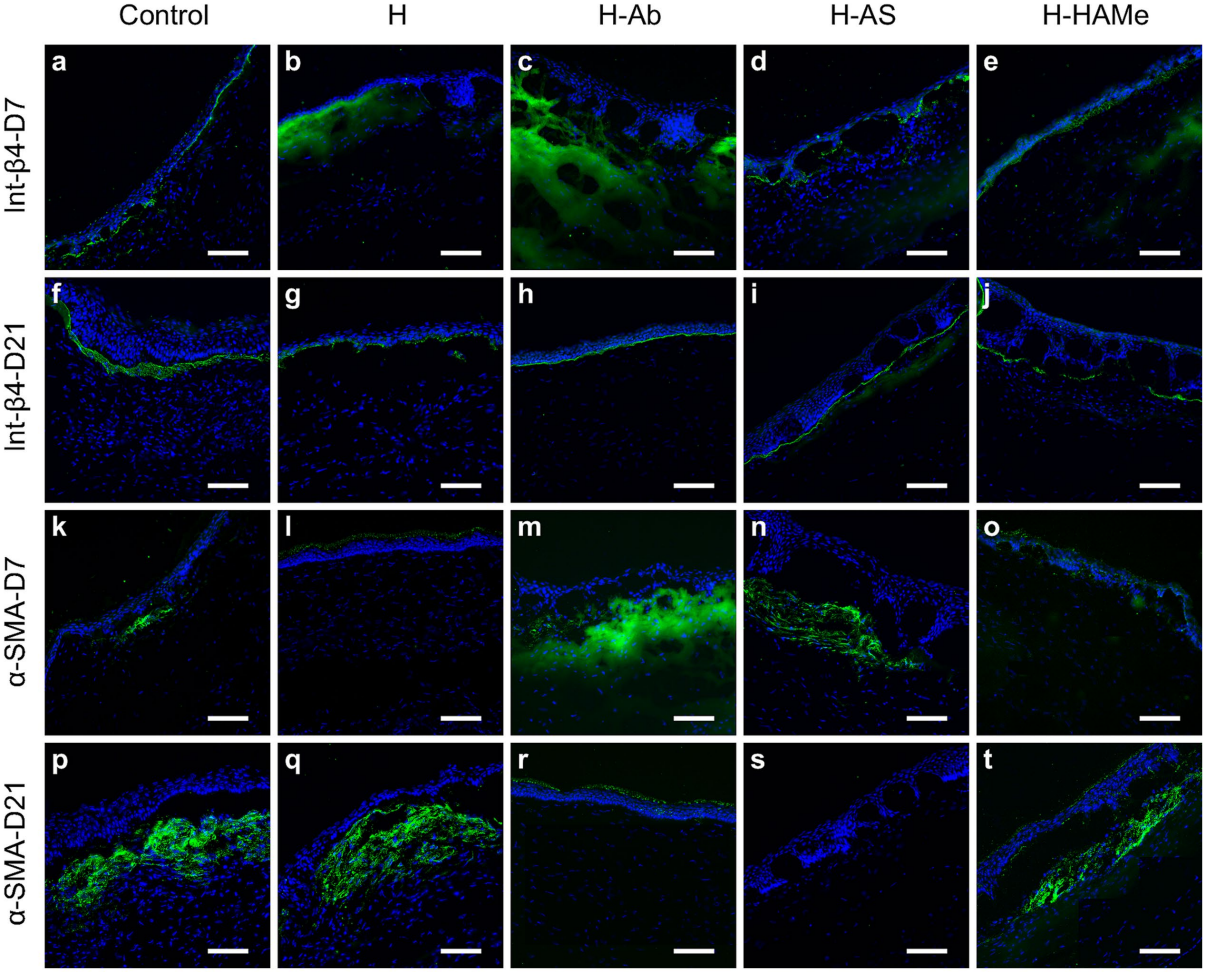
Protein expression of Integrin-β4 **(a–j)** and α-SMA (k–t) in the central cornea of rabbit corneas treated with artificial tears (Control) or hydrogels (H, H-Ab, H-AS, H-HAMe). Samples analyzed at 7 **(a–e,k–o)** and 21 days **(f–j,p–t)** post-surgery. Images at 20 × magnification; scale bars correspond to 100 μm.

Integrin β4 protein labeling, a cell-basement membrane adhesion molecule, showed discontinuity at 7 days but increased across all groups by day 21 (Figures 6a–j).

Immunohistochemical analysis of *α*-SMA, a marker of myofibroblast differentiation and fibrosis, revealed subepithelial α-SMA staining at day 7, with increased expression by day 21 in the control, H, and H-HAMe groups—evidence of progressive myofibroblast formation (Figures 6k–t). In contrast, H-Ab exhibited minimal staining, and H-AS showed no detectable α-SMA signal in the stroma (Figures 6r,s). At the RNA level, *α-SMA* expression was significantly reduced in H-Ab and H-AS groups at both day 7 (*p* = 0.0004) and day 21 (*p* = 0.0315 for H-Ab, *p* = 0.0012 for H-AS) compared to controls (Figures 5I,L). The H group at days 7 and 21 and the H-HAMe group at day 21 also showed reduced *α-SMA* levels, though these differences were not statistically significant (Figure 5L).

### 3.4 Eye irritation and inflammation

**Figure 7:**
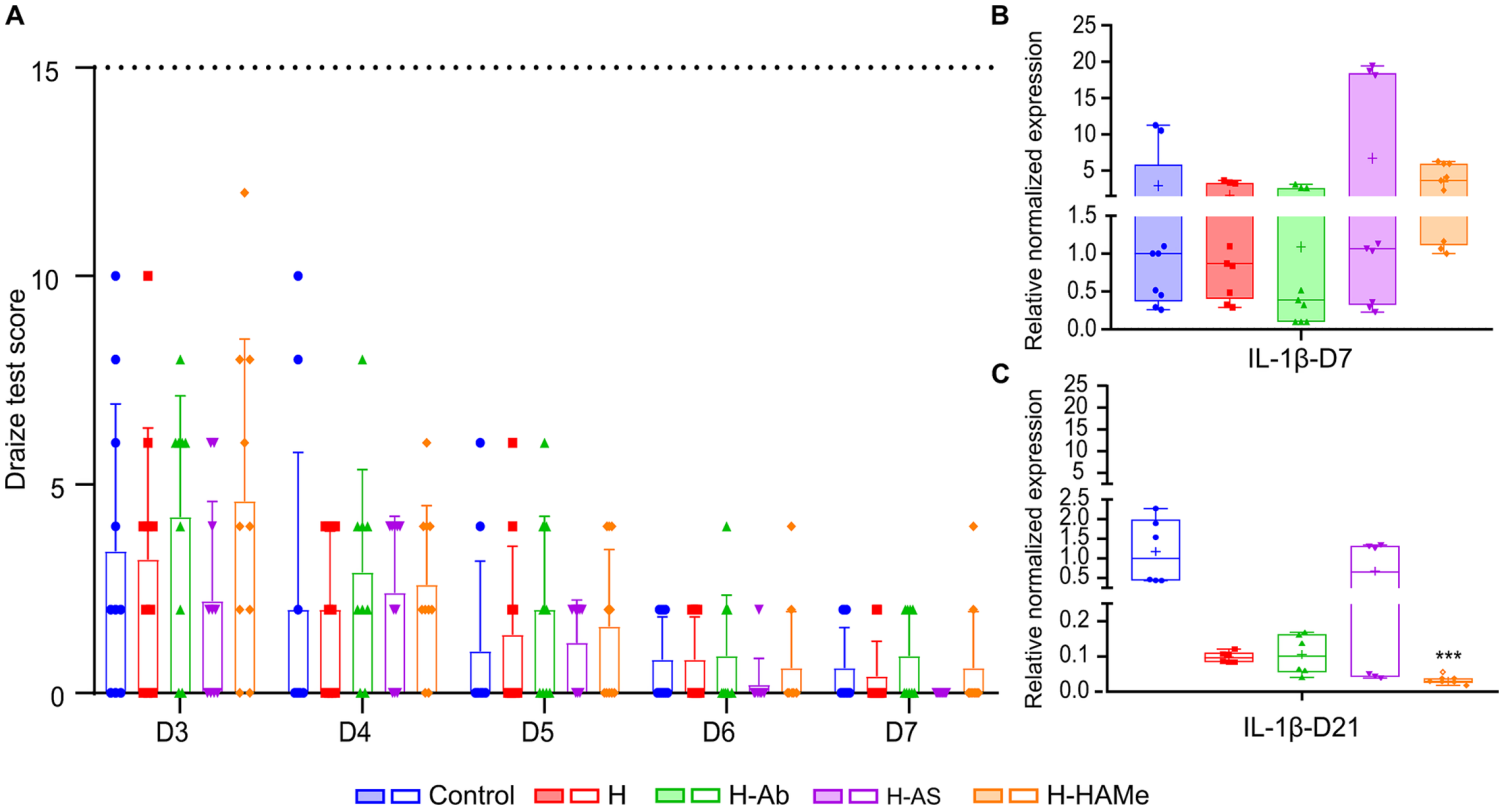
**(A)** Draize test scores from day 3 (D3) to day 7 (D7). Scores 2.5–15 (dotted line at y = 15) indicate minimal irritation; values above 15 are classified as mild or higher irritation. No significant differences were observed between samples. Relative normalized gene expression (ΔΔCq) of *IL-1β* in rabbit corneas 7 days **(B)** and 21 days **(C)** post-surgery. “+” sign is used to denote the mean value of each box plot. Significant differences (^∗^*p* < 0.05, ^∗∗^*p* < 0.01, ^∗∗∗^*p* < 0.001, ^∗∗∗∗^*p* < 0.0001) are indicated relative to the control treatment for each time point.

The Draize test showed minimal irritation across treatments by day 3, primarily due to surgery-induced redness, chemosis, or discharge (Figure 7A). H-AS exhibited the least irritant response among the tested formulations. Severe corneal opacity in one H-Ab-treated animal was excluded as an outlier. Symptoms improved by day 4, with control, H, and H-AS groups reaching non-irritative scores (0.6–2.5 points). H-Ab and H-HAMe achieved non-irritative levels by day 5. H-AS facilitated the fastest recovery, with irritation nearly resolved by day 6 (0.2 ± 0.63), and all groups scored 0 by day 7. No treatment exceeded 15 points, staying below the mild irritation threshold.

*IL-1β* levels measured by qPCR showed no significant differences at day 7 (Figure 7B). H-Ab reduced *IL-1β* expression by half, while H-HAMe showed increased levels compared to controls. By day 21, all hydrogel treatments led to a reduction in *IL-1β* levels, with significant decreases in H-HAMe (ΔΔCq: 0.05; *p* < 0.0001, Kruskal-Wallis test followed by Dunn’s multiple comparisons) and notable reductions in H-Ab (ΔΔCq: 0.18), and H (ΔΔCq: 0.17), indicating effective inflammation control (Figure 7C).

## 4 Discussion

In the context of tissue healing, replacement and regeneration are distinct concepts. Regeneration enables damaged tissue to fully restore affected parts to their original state by promoting new growth. Replacement refers to the healing of severely damaged area through the deposition of new abnormal tissue, it involves patching rather than restoring and it commonly implies scarring. The functionalized hydrogel aimed to promote stromal repair and tissue regeneration by facilitating the release of bioactive compounds. This non-surgical strategy sought to enable epithelial recovery, gradual stromal regeneration, and restoration of corneal transparency.

During the early experimental phase (days 3–5), the control group exhibited larger areas of epithelial closure compared to the hydrogel-treated groups, although some control corneas failed to fully close and occasionally reopened. Tarsorrhaphy appeared to accelerate epithelial defect closure. Additionally, the absence of the hydrogel as a physical barrier in the control group allowed for unhindered epithelial migration (45–47). Epithelial cells in the control group were able to migrate freely over the wound bed without encountering any physical barrier, unlike the hydrogel-treated corneas where the presence of the hydrogel could transiently modulate or slow cell migration. However, in some control cases this rapid closure appeared to be incomplete or unstable, resulting in partial wound reopening during the observation period. In contrast, although epithelial closure in the hydrogel-treated groups progressed more gradually, the histological images suggest a possible enhancement of epithelial–stromal adhesion in the hydrogel-treated corneas. This may reflect the hydrogel’s ability to provide a provisional matrix that supports more robust epithelial anchorage, potentially contributing to longer-term wound stability despite the slower initial closure rate.

Epithelial hyperplasia was observed in all corneas, likely representing a compensatory initial remodeling response to stromal loss to maintain corneal thickness and curvature (48). Proliferative activity, as indicated by increased numbers of Ki67-positive cells and elevated *Ki67* gene expression, was higher in all treatments by day 7, and especially in the control group.

All hydrogel treatments achieved complete re-epithelialization. H-AS demonstrated the fastest closure among hydrogel-treated groups from day 5, reflecting blood products’ epitheliothropic-enhancing effects (25–27, 49–52). H-HAMe also facilitated rapid closure, highlighting the pro-regenerative properties of amniotic membrane (AM) products (53–55). H-Ab exhibited slower initial closure and fewer cases closed by day 5 but achieved full re-epithelialization alongside H-AS. Infliximab, known for its anti-inflammatory properties, has been shown to support epithelial repair without compromising cell viability or phenotype (56, 57). The anti-inflammatory effects of Infliximab may permit autonomous epithelial repair in H-Ab treated corneas, leading to similar closure outcomes for H-Ab, H-AS, and H-HAMe.

Consistent ZO-1 labeling across groups at 7 and 21 days indicated a functional epithelium and prioritized barrier restoration during healing (52, 58, 59). Similarly, integrin β4, crucial for hemidesmosome formation and epithelial stability (60), showed discontinuous labeling at the wound site by day 7, with irregular staining in controls and gaps in hydrogel-treated corneas. By day 21, stronger integrin β4 staining indicated improved epithelial attachment and extracellular matrix integration.

CK15, a corneal epithelial progenitor marker (61, 62), was elevated in all corneas at day 21, indicative of limbal activation following injury. Although RNA analysis using whole sclerocorneal samples did not show statistically significant differences in *PAX6* or *p63* expression—likely due to dilution effects—a trend toward increased *p63* was noted at day 7 in H-AS and H-HAMe-treated corneas. Given that p63, particularly the ΔNp63 isoform, acts as a master regulator of epithelial stemness and self-renewal (63, 64), this early upregulation likely reflects activation of limbal epithelial stem/progenitor cells (LESCs) by growth factors present in HAMe and AS. This interpretation is in line with previous reports demonstrating that sPRGF and platelet-rich plasma formulations preserve stem/progenitor potential and enhance epithelial repair (25, 65, 66).

Correspondingly, AM-derived treatments have been shown to enhance LESC proliferation and stem cell marker expression while suppressing differentiation markers (67–69). The observed trends suggest that, in the presence of these bioactive agents, early stem cell activation precedes later differentiation, consistent with enhanced regenerative capacity.

At day 21, H, H-Ab, and H-AS treated groups displayed increased *PAX6* and decreased *CK3* expression. PAX6, a pivotal regulator of LESC fate and differentiation, is essential for maintaining the limbal niche and preventing transdifferentiation into non-corneal lineages (70, 71). Its upregulation in our hydrogel groups, as supported by *in vitro* data showing promotion of holoclone formation and suppression of differentiation markers (71), indicates preservation of stemness, likely mediated by the hydrogels’ modulation of inflammation and serving as a regenerative scaffold.

CD44, a transmembrane glycoprotein and HA receptor, is crucial for LESC adhesion, migration, and interaction within the niche, essential for maintaining the undifferentiated state and regenerative potential of LESCs (72, 73). Hydrogel-treated corneas exhibited generally elevated CD44 expression, with a statistically significant increase in the H group at day 7. Through CD44-HA interactions, hydrogels likely support sustained regenerative signaling, cytoskeletal remodeling, and epithelial repair (74–76). The use of 0.2% HA across all groups could have masked differences; however, the enhanced retention and local concentration of HA within the hydrogel vehicle may have reinforced CD44 activation in the H group, contributing to improved healing dynamics (77).

In summary, sequential elevation of p63 (early) and subsequent increases in PAX6 across hydrogel-treated groups, especially with H-AS and H-HAMe supplementation, reflect coordinated limbal activation and niche support. Notably, the hydrogels may also act as reservoirs for topically administered hyaluronic acid (HA), prolonging its residence time on the ocular surface and enhancing its biological effects. This HA retention could underlie the increased expression of CD44, a principal HA receptor, and may also contribute to the upregulation of PAX6. In contrast, the marked increase in p63 expression observed at day 7, particularly in the H-AS and H-HAMe groups, is more likely attributable to the presence of growth factors delivered via these specific hydrogel formulations. These findings suggest that the bioactive properties of hydrogels, including extended HA retention and localized delivery of growth factors, facilitate a mechanistic pathway of stem cell activation, niche preservation, and regenerative support in the injured cornea.

This study examined myofibroblastic responses in control and hydrogel-treated corneas after stromal injury, finding reduced *α-SMA* expression in hydrogel-treated samples. While an initial fibrotic response aids healing, excess myofibroblast-derived extracellular matrix can cause stromal opacity, requiring keratocyte-mediated reabsorption for transparency restoration. Hydrogel-treated corneas had significantly lower *α-SMA* levels at day 7 and 21, with H-Ab and H-AS showing the greatest reductions. Prior research demonstrated undiluted sPRGF eye drops reduce *α*-SMA expression, minimizing haze and scarring (25, 26). Gelatin in hydrogels likely influenced fibrosis, as GelMA hydrogels inhibit myofibroblast differentiation, prevent fibrosis, and maintain corneal properties (78). Increased GelMA hydrogel stiffness was associated with higher fibrosis-related gene expression and myofibroblast activity (79).

Immunofluorescence revealed α-SMA in the stroma and epithelium, indicating epithelial-mesenchymal transition (EMT) during corneal repair, consistent with prior studies, though the mechanisms remain unclear (80–82). Treatments for corneal scars often include corticosteroids (e.g., prednisolone, dexamethasone) to reduce inflammation and fibrosis, with severe cases requiring lenses or transplants (83–86). Hydrogel therapies demonstrated promise in reducing fibrosis and promoting stromal regeneration (87–90). While no treatment fully prevented fibrosis, hydrogels significantly reduced α-SMA expression, highlighting their potential to modulate early responses and reduce corneal opacification.

Draize scale assessments identified H-AS as the least irritating treatment, with H-Ab and H-HAMe causing mild irritation through days 3–5 but becoming non-irritating by day 6. Despite initial irritability, H-Ab reduced inflammation, as evidenced by *IL-1β* gene expression decreasing from a normalized median of 1 to 0.1 by day 21. Infliximab, a TNF-α inhibitor, has previously demonstrated efficacy in lowering *IL-1β* levels, inflammation, and fibrosis in corneal injuries (39). H-HAMe exhibited the strongest anti-inflammatory effect at day 21, likely due to HAMe-derived growth factors modulating cytokines by reducing TNF-*α* and IL-6 while increasing IL-10, promoting healing and reducing fibrosis and infection risk (91). HAM and its derivatives reduce corneal inflammation through IL-1ra and IL-10 secretion by amniotic epithelial cells (92). HAMe eye drops promote epithelial healing and limit conjunctivalization and vascularization in limbal stem cell deficiency (65), while HC-HA/PTX3 matrix component effectively suppresses inflammation, angiogenesis, and scarring (93).

H-Ab and H-HAMe hydrogels, which most effectively reduced *IL-1β* expression, initially caused greater clinical inflammation (e.g., chemosis and redness) as assessed by the Draize scale. In contrast, H-AS exhibited less redness and chemosis despite higher *IL-1β* expression at day 21. AS, enriched in factors that aid corneal repair, is effective in inflammatory conditions like severe dry eye disease (DED). Blood-derived drops, including AS and platelet products, surpass artificial tears due to regenerative properties (24, 94–99), though their efficacy varies with donor health, as elevated pro-inflammatory cytokines are observed in rheumatoid arthritis and Sjögren’s syndrome patients (100–102). To ensure consistency, rabbit sera were collected pre-surgery to avoid disease-related variations.

Considering the overall *in vivo* response, both functionalized and non-functionalized hydrogels enhanced wound healing by promoting re-epithelialization and significantly reducing the inflammatory and fibrotic response. In particular, H-AS facilitated the fastest epithelial closure, with reduced fibrosis and minimal irritability on the Draize scale. HAMe-functionalized hydrogels showed better anti-inflammatory effects but delayed epithelial closure and caused greater irritability. H-Ab effectively reduced IL-1β and α-SMA, demonstrating strong anti-inflammatory and antifibrotic properties, though epithelial closure was slower. Despite its higher initial irritability, it remained minimally irritating and achieved complete healing.

Among the limitations of the study, immunohistochemistry was applied qualitatively to support the qPCR findings, as image-based quantification proved technically challenging in this context. In addition, corneal transparency was assessed qualitatively through slit-lamp biomicroscopy performed by experienced ophthalmologists, combined with histological evaluation of fixed corneal sections. However, we acknowledge that a quantitative assessment of transparency—such as optical coherence tomography (OCT) or *in vivo* confocal microscopy—could have provided further insights into stromal remodeling and fibrotic changes.

Taken together, these findings highlight the therapeutic potential of functionalized hydrogels as bioactive platforms for corneal regeneration. This study set out to address the critical need for effective, biocompatible therapies that enhance corneal wound healing while minimizing inflammation and fibrosis—key challenges in the treatment of persistent epithelial defects and stromal injuries. By integrating hydrogel technology with clinically relevant bioactive components—including autologous serum, amniotic membrane extract, and infliximab—this work offers a novel comparative evaluation of advanced ophthalmic formulations within a controlled *in vivo* model.

The results demonstrate that each biofunctionalized hydrogel exerted beneficial effects on epithelial regeneration by promoting stemness and modulating fibrotic and inflammatory markers, albeit with differing kinetics and tissue responses. Importantly, our intention was also to emphasize that the hydrogel platform can be functionalized with different bioactive agents, thereby offering conceptual flexibility to tailor treatments to individual clinical needs and pathological contexts. These findings not only validate the regenerative potential of these formulations but also underscore the importance of selecting and combining functional components strategically to match specific therapeutic goals. This comparative approach, which is rarely explored in preclinical corneal research, provides new insights into the distinct roles of widely used ophthalmic biologics when integrated into a common hydrogel platform. Ultimately, the study advances the field of corneal regenerative medicine by establishing a foundational framework for the rational design and selection of multifunctional non-surgical biomaterial-based therapies.

## Data Availability

The original contributions presented in the study are included in the article/Supplementary material, further inquiries can be directed to the corresponding author/s.
