# Supplementary material for: Comparison of the regenerative potential of different functionalized gelatin-based hydrogels as fillers of rabbit corneal wounds
Source: Front Med (Lausanne). 2025 Sep 24;12:1667446. doi: 10.3389/fmed.2025.1667446 (PMC12504313; doi:10.3389/fmed.2025.1667446)
Supplement: Supplementary file 1 [file Data_Sheet_1.docx]

Supplementary Material

# Supplementary Figures and Tables

## Supplementary Tables

**Supplementary Table S1**. Experimental distribution and sample collection in the *in vivo* study.

|  | **Experiment E01 (Right eye)** | | | | | | **Experiment E02 (Left eye)** | | | | | |
| --- | --- | --- | --- | --- | --- | --- | --- | --- | --- | --- | --- | --- |
| Rabbit | Treatment | WH (D1-D7) | Draize (D1-D7) | H-E (D21) | IHC (D21) | qPCR (D21) | Treatment | WH (D1-D7) | Draize (D1-D7) | H-E (D7) | IHC (D7) | qPCR (D7) |
| 1 | H | Pilot study. Definition of the experimental conditions | | | | | Control | x | x | x | x |  |
| 2 | H-AS |  |  |  |  |  | H-HAMe | x | x |  |  | x |
| 3 | Control |  |  |  |  |  | H | x | x | x | x |  |
| 4 | Control |  |  |  |  |  | H-HAMe | x | x | x | x |  |
| 5 | H-Ab |  |  |  |  |  | H | x | x |  |  | x |
| 6 | H-AS |  |  |  |  |  | H-AS | x | x | x | x |  |
| 7 | H-HAMe |  |  |  |  |  | Control | x | x |  |  | x |
| 8 | H |  |  |  |  |  | H-AS | x | x |  |  | x |
| 9 | H-Ab |  |  |  |  |  | H-Ab | x | x | x | x |  |
| 10 | H-HAMe |  |  |  |  |  | H-Ab | x | x |  |  | x |
|  | **Experiment E03 (Right eye)** | | | | | | **Experiment E04 (Left eye)** | | | | | |
| Rabbit | Treatment | WH (D1-D7) | Draize (D1-D7) | H-E (D21) | IHC (D21) | qPCR (D21) | Treatment | WH (D1-D7) | Draize (D1-D7) | H-E (D7) | IHC (D7) | qPCR (D7) |
| 11 | Control | x | x |  |  | x | H-Ab | x | x | x | x |  |
| 12 | H | x | x | x | x |  | H-HAMe | x | x |  |  | x |
| 13 | H-Ab | x | x | x | x |  | H | x | x | x | x |  |
| 14 | H-HAMe | x | x |  |  | x | H | x | x |  |  | x |
| 15 | H-AS | x | x |  |  | x | H-HAMe | x | x | x | x |  |
| 16 | Control | x | x | x | x |  | H-AS | x | x |  |  | x |
| 17 | H-AS | x | x | x | x |  | Control | x | x | x | x |  |
| 18 | H-Ab | x | x |  |  | x | H-AS | x | x | x | x |  |
| 19 | H-HAMe | x | x | x | x |  | Control | x | x |  |  | x |
| 20 | H | x | x |  |  | x | H-Ab | x | x |  |  | x |
|  | **Experiment E05 (Right eye)** | | | | | | **Experiment E06 (Left eye)** | | | | | |
| Rabbit | Treatment | WH (D1-D7) | Draize (D1-D7) | H-E (D21) | IHC (D21) | qPCR (D21) | Treatment | WH (D1-D7) | Draize (D1-D7) | H-E (D7) | IHC (D7) | qPCR (D7) |
| 21 | H-HAMe | x | x | x | x |  | H-Ab | x | x | x | x |  |
| 22 | H | x | x |  |  | x | Control | x | x |  |  | x |
| 23 | H-AS | x | x |  |  | x | H-Ab | x | x |  |  | x |
| 24 | Control | x | x |  |  | x | H-AS | x | x | x | x |  |
| 25 | H-AS | x | x | x | x |  | H-HAMe | x | x |  |  | x |
| 26 | H-Ab | x | x |  |  | x | H-AS | x | x |  |  | x |
| 27 | H | x | x | x | x |  | H | x | x | x | x |  |
| 28 | H-Ab | x | x | x | x |  | Control | x | x | x | x |  |
| 29 | H-HAMe | x | x |  |  | x | H-HAMe | x | x | x | x |  |
| 30 | Control | x | x | x | x |  | H | x | x |  |  | x |

Ab: Antibody; AS: Autologous serum; D: days post-surgery of the study-eye; H: Hydrogel; HAMe: Human amniotic membrane extract; H-E: Hematoxylin-eosin; IHC: Immunohistochemistry; qPCR: quantitative Polymerase Chain Reaction analysis; WH: Wound healing.

**Supplementary table S2**. P-Values for wound healing analysis.

| **Day** | **P value** |
| --- | --- |
| D3 | 0.0168 |
| D4 | 0.0016 |
| D5 | <0,0001 |
| D6 | <0,0001 |
| D7 | <0,0001 |

**Supplementary table S3**. p-Values for qPCR analysis.

|  | **D7** | **D21** |
| --- | --- | --- |
| αSMA | <0,0001 | <0,0001 |
| CD44 | 0.0059 | 0.0004 |
| CK3 | <0,0001 | <0,0001 |
| IL1B | <0,0001 | <0,0001 |
| Ki67 | <0,0001 | <0,0001 |
| p63 | 0.0001 | 0.0078 |
| PAX6 | <0,0001 | <0,0001 |
